# The consequences of artemisinin resistance in *falciparum* malaria

**DOI:** 10.1186/1475-2875-11-S1-O36

**Published:** 2012-10-15

**Authors:** Nicholas J White

**Affiliations:** 1 Mahidol-Oxford Research Unit, Faculty of Tropical Medicine, Mahidol University, Bangkok, Thailand

## 

Artemisinin resistance in *Plasmodium falciparum* has emerged in South East Asia. Evidence of resistance in Western Cambodia was documented over six years ago, and last year clear evidence of resistance was reported on the Thailand- Myanmar border. As artemisinin derivatives are the cornerstone of antimalarial treatment both for uncomplicated and severe malaria, their loss will increase morbidity and mortality, and could derail current containment and elimination efforts. In the treatment of severe *falciparum* malaria artesunate reduced mortality by approximately one third compared with quinine. This resulted from greater parasiticidal activity against circulating ring stage parasites, the very property that is lost in artemisinin-resistance. If we have to return to quinine for the treatment of severe malaria, mortality will rise again. In the treatment of uncomplicated malaria artemisinin resistance results in slower parasite clearance and consequently slower therapeutic responses. Times to recovery are slower, treatment failure rates higher, and transmissibility of the treated infection greater. The reduced antiparasitic effect means that the partner drug now has toeliminate a greater proportion of the infecting parasite biomass, and so there is a greater probability of selecting for partner drug resistance. The future of artemisinin resistance is uncertain; the mechanism underlying resistance has yet to be elucidated. With present levels of resistance artemisinins are still efficacious, albeit much more slowly than before. Whether higher levels of resistance can and will occur is uncertain. Nevertheless, given that previous pandemic spread of antimalarial resistance from South-East Asia killed millions, containing this threat is of the highest global health priority.

